# Micronutrient Powders Combined With Malaria Chemoprevention to Improve Anaemia and Cognitive Function in Early Childhood in Mali: A Cluster‐Randomised Trial

**DOI:** 10.1111/mcn.70033

**Published:** 2025-05-20

**Authors:** Niélé H. Diarra, Yahia Dicko, Natalie Roschnik, Modibo Bamadio, Michael Boivin, Yvonne Griffiths, Rebecca Jones, Sham Lal, Helen Moestue, Hamidou Niangaly, Lauren Pisani, Renion Saye, Kalifa Sidibe, Karla Smuts, Nia‐An Philippe Thera, Josselin Thuilliez, Hans Verhoef, Moussa Sacko, Siân E. Clarke

**Affiliations:** ^1^ Save the Children International Bamako Mali; ^2^ Save the Children Federation Inc. Washington District of Columbia USA; ^3^ Department of Psychiatry and Department of Neurology and Ophtalmology Michigan State University East Lansing Michigan USA; ^4^ School of Education University of Leeds Leeds UK; ^5^ University College London London UK; ^6^ Faculty of Infectious Tropical Diseases London School of Hygiene and Tropical Medicine London UK; ^7^ Malaria Research and Training Center University of Sciences, Techniques and Technologies Bamako Mali; ^8^ Institut National de Recherche en Santé Publique Ministère de la Santé et du Développement Social Bamako Mali; ^9^ Centre d'Economie de la Sorbonne CNRS – Universite Paris 1 Paris France; ^10^ Division Human Health and Nutrition Wageningen University Wageningen The Netherlands

**Keywords:** anaemia, chemoprevention, child development, cognition, dietary supplements, fortification, malaria, micronutrients, powders

## Abstract

A cluster‐randomised controlled trial was conducted in 60 communities in southern Mali to evaluate the impact of micronutrient powders (MNP) combined with seasonal malaria chemoprevention (SMC) on anaemia (primary endpoint), *Plasmodium* infection, stunting and cognitive function in children < 5 years. The 60 communities were randomly allocated to the intervention or control arm, and cross‐sectional biomedical and cognitive surveys were conducted after 1 and 3 years in a random sample of 3 and 5 years olds (1052 and 1081 children, respectively). All children aged 3–59 m in intervention and control communities received two rounds of SMC each year during the peak malaria season, and in intervention communities, all children aged 6–59 m additionally received 4 months of daily MNP after the peak malaria season. Despite a high baseline prevalence of anaemia and good fidelity to intervention, this trial found no evidence of impact on study outcomes. The prevalence of anaemia was similar in both arms for both age groups after 1 and 3 years of intervention—after 3 years, the prevalence of anaemia amongst 3‐year olds was 57.6% in the intervention arm versus 60.1% in the control group (*p* = 0.352). For 5‐year olds, it was 51.3% and 53.0%, respectively (*p* = 0.607). No effect was observed on stunting or cognitive function either.

## Summary

- Around 60% of children under 5 years in Africa are anaemic due to iron deficiency and disease. Home fortification with micronutrient powders (MNP) and seasonal malaria chemoprevention (SMC) are recommended interventions to prevent anaemia and malaria.
- A cluster randomised trial in 60 rural communities in Southern Mali found that MNP, combined with SMC, had no impact on anaemia, stunting or cognitive development despite relatively high adherence and a high burden of anaemia and malaria.
- While MNP are effective in most contexts, they are not effective in every context. More research is needed to assess MNP's capacity to prevent anaemia in a wider range of settings—addressing both the biological mechanisms that might hinder absorption, as well as the sociocultural aspects for maximal utilisation.

## Graphical abstract

Globally nearly 40% of children under 5 years old are anaemic, which affects their ability to grow and develop. A cluster randomised trial was conducted in 60 communities in Southern Mali to evaluate the added impact of home fortification with micronutrient powders, delivered alongside seasonal malaria chemoprevention, on anaemia, malaria infection, stunting and cognitive function in early childhood.

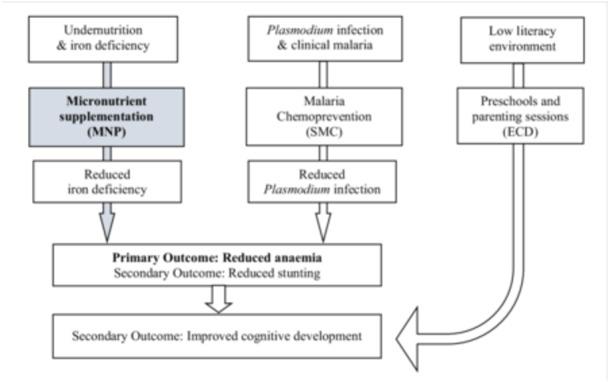

## 1 Introduction

Forty percent of children under 5 years globally are anaemic, as many as 60% in Africa (WHO 2021). Anaemia, which is defined by low haemoglobin concentration, is largely invisible but affects children's ability to grow and develop to their full potential. There is strong evidence that iron‐deficiency anaemia results in impaired cognitive development in young children (Grantham‐McGregor and Ani 2001). The main causes of anaemia are multiple, including iron deficiency, other micronutrient deficiencies, and acute and chronic infections such as malaria (WHO 2017, 2022; Safiri et al. 2021). In 2011, home fortification with micronutrient powder (MNP) was added to the list of recommended interventions to improve iron status and reduce anaemia risk among children aged 6–23 months where the prevalence of anaemia was 20% or higher; later updated to include children aged 2–12 years (WHO 2011, 2016). In 2012, WHO recommended seasonal malaria chemoprevention (SMC)—up to four monthly rounds of antimalarial drug combination treatment targeting all children aged 3–59 months during the peak malaria season—to prevent malaria‐related morbidity and mortality (WHO 2012). SMC has also been shown to reduce anaemia and improve growth (Wilson 2011; Ntab 2007).

**Figure 1:**
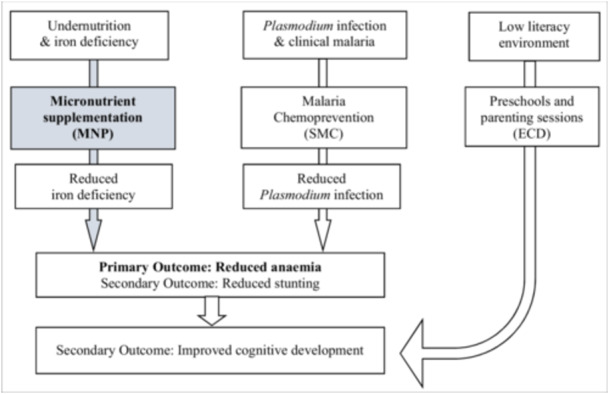
Theory of change: Expected benefits of micronutrient powder supplementation trial, in combination with other supporting interventions.

Many studies have evaluated the impact of MNP worldwide, with three recent meta‐analyses concluding that MNP reduce the risk of anaemia in children (De‐Regil et al. 2017; Tam et al. 2020; Suchdev et al. 2020). However, only a few of these trials were conducted in low‐income settings, and none to date have evaluated the impact of MNP combined with malaria chemoprevention on nutrition, health and child development outcomes. Given the high prevalence of malaria, anaemia and poor child development in Mali and Sikasso region (INSTAT 2014, 2016), combining MNP with SMC would be expected to have a multiplying effect on children's health, nutrition and cognitive development. The aim of the trial was to evaluate the combined impact of MNP and SMC on a range of health, nutrition and child development outcomes at a point when both interventions had been newly recommended by WHO but had not yet been implemented in Mali. The underlying supposition was that combining MNP with SMC would jointly prevent anaemia by addressing the two main causes of anaemia (iron deficiency and malaria infection), which would in turn improve child growth, reduce stunting and improve cognitive development (Figure 1).

This paper reports the results from a cluster randomised trial amongst preschool children to evaluate the impact of MNP, combined with SMC, delivered through community preschools, on anaemia, *Plasmodium* infection, stunting and cognitive function.

## 2 Methods

### 2.1 Study Area and Population

The study was undertaken in the Sikasso region of southern Mali between 2013 and 2016, an area of high disease burden where 86% of children under 5 years were anaemic, 93% had malaria, 40% were stunted in 2012 (INSTAT 2014) and 38% of children were not meeting key child development milestones in 2015 (INSTAT 2016).

### 2.2 Trial Design and Allocation to Intervention

The study was an open‐label, stratified cluster‐randomised controlled trial implemented in 60 rural communities in Sikasso and Yorosso districts between July 2013 and July 2016. Sixty communities were randomly selected from a total of 82 villages with nongovernmental organisation (NGO)‐supported preschools to be part of the study. Villages were stratified by years of preschool operation and randomly allocated within strata to the intervention or control arm using a computer‐generated random list by an independent statistician (R.J.) with no prior knowledge of the study area. The unit of randomisation was the village. All families resident in study villages with young children under 5 years of age were eligible to receive the interventions, irrespective of preschool enrolment.

### 2.3 Design of the Intervention

In the first phase of the trial (October 2013–July 2014), all children under 5 years resident in the 30 villages in the intervention arm received MNP and SMC (see protocols below), and children in the control villages received neither. In the following 2 years of the trial (October 2014–July 2016, phase 2), all children under 5 years in the 30 intervention villages continued to receive MNP. However, since SMC became a national programme led by the Ministry of Health in 2014, SMC was scaled up to all 60 villages (including the control arm). Throughout all 3 years of the trial, all children, in both arms, benefitted from routine preventative services, for example, deworming and vitamin A supplementation, and NGO‐supported ECD interventions (parenting education and preschools).

*The MNP protocol was as follows:* All children aged 6–59 months received a sachet of MNP daily for four consecutive months each year (January–April 2014, January–April 2015 and January–April 2016), a total of 120 sachets containing 10 mg iron per year. The WHO recommends 90 sachets containing 10–12.5 mg of elemental iron to be given over a 6‐month period (WHO 2011, 2016). However, to avoid the provision of iron‐containing supplements during the malaria season in this highly endemic area for malaria, the annual dose of MNP was given over a shorter 4‐month period during the dry season when malaria transmission is minimal. MNP were added to a small quantity of children's morning porridge by caregivers on a daily basis. The caregivers were trained on how to administer MNP through infant and young child feeding and parenting education sessions and cooking demonstrations. MNP deliveries and materials were designed based on formative qualitative research, described in more detail elsewhere (Roschnik et al. 2019).

*The SMC protocol was as follows:* All children aged 3–59 months received two rounds of SMC during the months of peak malaria transmission (in October and November each year in 2013, 2014 and 2015), and before the MNP intervention. While the WHO recommendation was to provide SMC monthly for up to four rounds per year, for budget and logistical reasons, only two rounds were provided. SMC (sulfadoxine‐pyrimethamine, combined with amodiaquine) was provided by trained health workers at the preschool or other central site in the community.

### 2.4 Impact Evaluation

**Figure 2:**
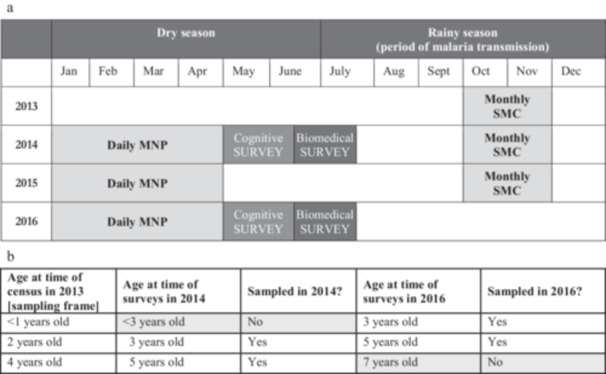
(a) Timing of the MNP and SMC distributions and evaluation activities. (b) Age of children sampled in cross‐sectional surveys in 2014 and 2016. MNP, micronutrient powder; SMC, seasonal malaria chemoprevention.

The impact of the interventions on cognitive and biomedical outcomes was evaluated through two cross‐sectional surveys at the end of the dry season, the first in May–July 2014 (after 7–9 months of intervention) and the second in May–July 2016 (after 2 years and 7–9 months of intervention). Both surveys were conducted 3–9 weeks after the last round of MNP distributions. At the time of the 2014 survey, intervention villages had received the combined SMC + MNP intervention for 7–9 months, and control villages had received neither intervention. Shortly after the 2014 survey, SMC was extended to include all villages in the trial as part of the national scale‐up of SMC by the Ministry of Health. Thus, at the time of the 2016 survey, the intervention villages had received the combined MNP + SMC intervention for nearly three consecutive years, and control villages had received SMC only for nearly 2 years (see Figure 2).

#### 2.4.1 Biomedical Outcomes

Data on nutritional status and other health outcomes were collected in June–July, in 2014 and 2016, by a survey team blind to the intervention status of study villages. Biomedical outcomes were assessed in two age groups of children—those aged 3 years and those aged 5 years at the time of the survey. Finger prick blood samples were obtained from each child to assess the prevalence of anaemia and infection with malaria parasites. The primary endpoint was anaemia, defined as a haemoglobin concentration of less than 110 g/L, assessed using a portable photometer (Hemocue Hb 301, Angleholm, Sweden). Malaria parasite infection was defined as the presence of asexual stages (trophozoites and/or schizonts) of any *Plasmodium* species in a thick blood film. Slides were declared negative after examination of 100 negative high‐powered fields. Microscopy was repeated on a 10% random sample of slides for quality control. Other secondary outcomes included prevalence of moderate‐to‐severe anaemia (haemoglobin less than 100 g/L), prevalence of stunting (height‐for‐age *Z* score < − 2SD), prevalence of underweight (weight‐for‐age *Z* score < − 2SD), prevalence of acute malnutrition (weight‐for‐height *Z* score < − 2SD in 3‐year olds only, BMI‐for‐age Z score < ‐2SD in 5‐year olds) and haemoglobin concentration. Malaria parasite density and malaria infectiousness (the presence of gametocytes of any *Plasmodium* species) were also measured.

#### 2.4.2 Cognitive Outcomes

Cognitive‐linguistic and executive function (foundation skills for early literacy and numeracy) were assessed in children in May 2014 and 2016 in the same individuals as those examined in the biomedical surveys. Two sets of age‐appropriate cognitive tests were assembled, and each battery was piloted and adapted to the local context, culture and language, before use. The tests used for each age group and the skills assessed by each are summarised in Tables 3a, 3b and S1. Children were assessed individually by a team of district education staff blind to the intervention status of study villages and trained using standardised instructions. All testing was performed in the child's mother tongue (Bambara, Shenara or Mamara), except in a minority of children from other ethnic groups. A structured household questionnaire was administered with the child's parent/guardian to record data on the home environment and other covariates, including parental education, socioeconomic status, preschool enrolment, levels of engagement with MNP, SMC and parenting interventions and recall of play activities.

#### 2.4.3 Sample Size

Assuming an anaemia prevalence of 50% in the control group and an intraclass correlation (ICC) of 0.08, a minimum sample size of 14 children per community in a trial with 30 clusters per arm was estimated to provide 80% power to detect a reduction in anaemia of at least 28% at 5% level of significance. The observed ICC for anaemia in the 2014 survey was 0.08 in 3‐year‐old children and 0.06 in 5‐year‐old children. For cognitive‐linguistic outcomes, where an ICC of 0.10 was assumed, a sample size of 16 children in each age group sampled per community were estimated to provide 80% power to detect an effect size of at least 0.30 at 5% level of significance. The observed ICC in 2014 was 0.09 (ranging from 0.05 to 0.16 for individual tasks within the cognitive battery).

#### 2.4.4 Sampling Procedures

A household census conducted in 2013 was used as a sampling frame for both the 2014 and 2016 surveys. Two age groups (3‐ and 5‐year olds) were surveyed at each time point and assessed for biomedical and cognitive outcomes to assess the impact of exposure to the intervention at different stages of early childhood. For each age group, a random sample of 20 children from each village was selected to be surveyed. Figure 2b shows the sampling strategy for the groups of children sampled in the cross‐sectional surveys in 2014 and/or 2016.

All children surveyed at age 3 years old in 2014 and who were still residents in the same village in 2016 were eligible for inclusion in the 2016 survey at age 5 years old. In villages where there was an insufficient number of children surveyed in 2014 who were still residents in 2016, additional children were recruited prior to the 2016 survey. Replacements were selected at random from the (household census) list of children resident in the village in 2013 to ensure that they were resident and exposed to the intervention throughout the 3‐year trial duration. Of the 1577 children aged 3 years at the time of the surveys in 2014, a total of 1437 (91%) were successfully contacted and resurveyed in 2016. The characteristics of those resurveyed in 2016 and those no longer resident in the study villages were similar (Table S2). There were no documented refusals or withdrawal of consent. The 5‐year olds surveyed in 2014 and the 3‐year olds surveyed in 2016 were only surveyed once since they were either too old or too young at the time of the other survey. The latter group of children born in January–July 2013 (too young to be included in the 2014 survey) had received the interventions throughout their eligible lifetime from 3 to 6 months of age onwards up until the age of 3 years when they were surveyed in 2016. Informed consent was obtained from the child's parent or guardian before inclusion in each of the surveys.

#### 2.4.5 Statistical Analysis

Data were analysed by intention‐to‐treat, using methods appropriate for a cluster‐randomised trial (Hayes and Moulton 2009). Statistical analyses were performed separately on data from children in each of the two age groups. Logistic regression models, accounting for random effects of clustering, were used to compare the prevalence of binary biomedical outcomes (anaemia, stunting and wasting) between the two arms. Analyses were conducted separately for each survey. For continuous measures, such as haemoglobin concentration and cognitive outcomes, linear regression models were employed. Since cognitive score data were often not symmetrical, a bootstrap technique was used to estimate 95% confidence intervals. To minimise statistical concerns of multiple comparisons, no more than 10 outcomes (including the primary outcome) are considered for formal statistical testing at the 5% level. Adjusted analyses for all outcomes included the following covariates defined a priori: sex, language spoken in the home, maternal literacy and household socioeconomic score. For health outcomes, adjusted analyses were additionally controlled for *Plasmodium* infection status at the 2016 survey; whilst the final adjusted models for cognitive parameters additionally included preschool enrolment. Exploratory sub‐group analyses were conducted for a small set of prespecified characteristics. Anthropometric indices were calculated using WHO Anthro software (WHO 2019) and data were analysed using Stata v14.

#### 2.4.6 Cost Analysis

A cost analysis of the MNP intervention was conducted from a provider perspective, using 1‐year time horizon, to estimate the total costs of programme delivery and management and the average unit cost per child treated. Since SMC was implemented across both arms from 2014, costings covered only MNP. Data collection was carried out following a top‐down approach, from project coordination to financial management, and costs were calculated using an ingredient approach, which identifies all the inputs, their quantity and value (Drummond et al. 2005; Barberton and Carter 2016). For inputs with a lifespan of more than 1 year, annualised costs were used. Univariate sensitivity analysis was used to assess the robustness of the resultant cost analysis to variation in unit costs and to determine how changes in some categories could affect the total cost (Jain et al. 2011; Fiedler and Puett 2015).

### 2.5 Ethics

Ethical approval for (a) the trial and (b) follow‐up after 3 years of implementation was obtained from the Comite d'Ethique de l'INRSP, Ministry of Health, Mali (06/13/INRSP‐CE and 06/13/INRSP‐CE, respectively) and the London School of Hygiene & Tropical Medicine (LSHTM) ethics committee, UK (6489; 11,335). In July 2013, community meetings were held with parents and local community representatives to explain the purpose of the study and procedures to be followed (including randomisation of communities), after which communities were offered the choice to participate in the trial. Community meetings were repeated in May 2014 and May 2016 to obtain written informed consent from the parents of each child selected to participate in the surveys.

### 2.6 Trial Registration

The trial was not prospectively registered because both co‐principal investigators mistakenly believed the registration had been completed in 2013. This oversight reduces methodological transparency and poses a theoretical risk of selective‐reporting bias. However, the null findings make it improbable that the lack of registration materially influenced the analyses or conclusions.

## 3 Results

### 3.1 Sample Population

At the time of the final evaluation in May–July 2016, children resident in intervention villages were eligible to have received MNP annually for 3 years as well as SMC for 3 years, whereas children resident in control villages would only have received SMC for two malaria seasons through the national campaign and no MNP. Among children aged 3 years in 2016, a total of 1082 were assessed for biomedical outcomes (538 intervention and 514 control), and 1081 for cognitive outcomes (543 intervention and 538 control); whilst amongst 5‐year‐old children, 1081 (530 intervention and 551 control) were assessed for biomedical outcomes, and 1115 (551 intervention and 564 control) for cognitive outcomes.

In 2014, after 1 year of intervention, 1105 (550 intervention and 555 control) 3‐year olds and 1033 (522 intervention and 511 control) 5‐year olds were assessed for biomedical outcomes. Since around a third of the 2014 household survey cognitive data were lost due to a computer virus, 2014 biomedical and cognitive outcomes could not be adjusted for the family socioeducational environment and are thus not reported in this paper. There were no documented refusals or withdrawal of consent. Analysis was carried out separately for each time point. This paper primarily reports the 2016 survey results (as the final evaluation after three successive years of intervention), with the corresponding unadjusted 2014 biomedical survey results presented in the supporting materials.

### 3.2 Characteristics of Study Population

In this rural population, the main source of income was subsistence farming and parental literacy was generally low (Table 1). The characteristics of children surveyed in 2016 were broadly similar across the two arms (Table 1). Reported preschool enrolment was lower amongst 3‐year olds and was substantially higher in the intervention arm. Parental reports of malaria prevention measures (use of insecticide‐treated net and receipt of SMC) were also higher in the intervention arm for both age groups, though these differences were slight. Reports of types of foods eaten, home literacy environment and parent–child interactions in the week before the survey were broadly similar (Table S3).

**Table 1:** Characteristics of 3‐ and 5‐year‐old children in each study arm assessed in 2016, after 3 years of intervention.^a^

	3‐year olds				5‐year olds			
	*n* = 1136				*n* = 1163			
	Intervention		Control		Intervention		Control	
Characteristics of children^a^	*n* = 583		*n* = 553		*n* = 590		*n* = 573	
	%	*n*/*N*	%	*n*/*N*	%	*n*/*N*	%	*n*/*N*
Male child	48.7	282/579	53.2	294/553	52.1	306/588	54.1	308/569
*Main language spoken at home*								
‐ Bambara	34.9	201/576	34.0	187/550	35.1	207/589	31.5	180/572
‐ Shenara	50.2	289/576	48.4	266/550	50.1	295/589	50.9	186/572
‐ Mamara	6.4	37/576	4.6	25/550	5.9	35/589	5.2	30/572
‐ Other ethnicity	8.5	49/576	13.1	72/550	8.8	52/589	12.5	71/572
Maternal literacy	16.3	93/569	18.5	101/546	18.4	108/586	13.6	76/560
Paternal literacy	36.2	205/567	32.4	175/540	33.6	193/575	32.9	184/560
*Income:* Subsistence agriculture	94.8	545/575	92.2	506/549	93.7	551/589	90.6	518/572
*Standard of housing*								
Metal roof	92.9	534/575	93.4	513/549	93.4	550/589	92.7	530/572
Tile/concrete floor	35.3	203/576	43.1	237/550	34.8	205/589	36.0	206/572
Brick/concrete walls	4.2	24/573	7.6	41/541	3.4	20/585	5.1	29/569
‐ Solar/electric lighting	80.7	465/576	79.6	438/550	83.0	488/588	82.0	449/552
*Parent/caregiver report*								
‐ Variety in child's diet limited by financial resources in last 4 weeks	39.5	223/565	36.7	199/542	37.1	215/579	35.5	199/560
‐ Child ever went go to bed hungry in last 4 weeks	8.4	48/571	6.6	36/547	7.3	43/586	5.1	29/569
‐ Child enroled in preschool	41.5	238/574	25.4	139/548	55.0	323/587	47.9	273/570
‐ Child slept under insecticide‐treated net last night	92.2	528/573	89.4	489/547	93.0	547/588	87.2	497/570
‐ Child received SMC in 2015	96.1	537/559	90.1	473/525	97.6	563/577	92.2	505/548
‐ Child ever given MNP	81.0	451/557	17.6	89/505	79.6	460/578	21.2	112/528

Abbreviations: MNP, micronutrient powder; SMC, seasonal malaria chemoprevention.

^a^ Data were collected from parental/caregiver interviews 1 month before biomedical and cognitive assessments; some children were absent on the day of the subsequent biomedical or cognitive surveys (no outcome data available).

### 3.3 Use of MNP

Reported use of MNP exceeded 80% in the intervention arm in both age groups (Table 1). Monitoring data and qualitative research in intervention villages, reported in detail elsewhere (Roschnik et al. 2019), were also indicative of good fidelity to intervention, finding the interventions to be well accepted by parents, preschool staff, women's groups and community leaders, and the MNP generally well liked by the children (Table S4). It is important to note that reported “ever use of MNP” was also reported for 18% and 21% of 3‐ and 5‐year olds in the control arm.

### 3.4 Age‐Specific Prevalence of Health Outcomes in 2014 and 2016

**Figure 3:**
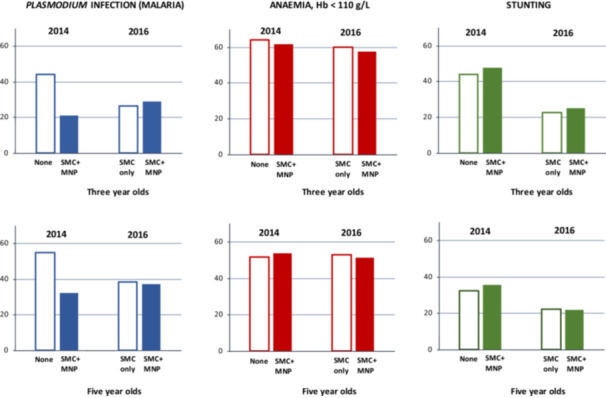
Prevalence of biomedical outcomes in children aged 3 and 5 years in 2014^a^ and 2016^b^ (white bars show prevalence in the control arm; filled bars show prevalence in the intervention arm). Interventions received: SMC, seasonal malaria chemoprevention. MNP, home fortification with micronutrient powders. ^a^By the time of the survey in 2014, children resident in intervention villages were eligible to have received both SMC and MNP for one year, whereas children resident in control villages had received neither. ^b^By the time of the survey in 2016, children resident in intervention villages were eligible to have received MNP annually for 3 years as well as SMC for three malaria seasons, whereas children resident in control villages would only have received SMC for two malaria seasons through the scaled‐up national campaign.

More than 60% of children surveyed at age 3 years in June 2014 were anaemic (Hb < 110 g/L); and close to a third with moderate‐to‐severe anaemia (Hb < 100 g/L). The prevalence of anaemia was lower in the 5‐year olds but still exceeded 50%. The age‐specific prevalence of anaemia did not change over time, remaining similar between the intervention and control arms, both in 2014 after 1 year of intervention and in 2016 after 3 years of intervention (Figure 3). In contrast, the age‐specific prevalence of *Plasmodium* infection in June 2014 was substantially reduced after 1 year of the combination intervention (SMC + MNP) in intervention villages compared with control villages receiving neither intervention, in both age groups; *p* < 0.001, respectively (Figure 3 and Tables S5a and S5b). After the rollout of SMC to all study villages in line with national policy, the prevalence of *Plasmodium* infection also decreased in the control arm. By 2016, there was little difference in age‐specific *Plasmodium* infection prevalence between children in the intervention villages (SMC + MNP) and control villages (SMC only; Figure 3). The age‐specific prevalence of stunting also declined over time, though remained similar across the intervention and control arms at both time points.

### 3.5 Effect of Intervention on Biomedical Outcomes After 3 Years of Intervention

Biomedical outcomes measured in July 2016 in each age group were compared between the two randomised arms of preschool villages after 3 years of intervention. Despite being eligible to receive MNP since weaning and thus exposed to the MNP since infancy, the prevalence of anaemia (Hb < 110 g/L; primary outcome) in 3‐year‐old children was similar between children receiving MNP and those who did not (57.6% intervention arm vs. 60.1% control arm, respectively; aOR 0.84 [95% CI 0.59–1.21]; *p* = 0.352); likewise moderate‐to‐severe anaemia (Hb < 100 g/L; 27.1% vs. 31.3%; aOR 0.70 [95% CI 0.47–1.04]; *p* = 0.081, Table 2a). Mean haemoglobin concentration in 3‐year‐olds postintervention was also similar in intervention and control arms: 105.9 and 104.4 g/L, respectively. Among 5‐year‐old children, likewise, there was no difference in the prevalence of anaemia (51.3% intervention vs. 53.0% control; aOR 0.90 [95% CI 0.60–1.35]; *p* = 0.607, Table 2b), nor in mean haemoglobin levels (108.0 and 107.8 g/L, respectively). Geometric mean serum ferritin was higher among 5‐year‐old children in the intervention arm (90.02 μg/L vs. 74.44 μg/L control; adjusted difference 0.18 [95% CI 0.07–0.30]; *p* = 0.002, Table 2b); no difference was observed in 3‐year‐old children. All anthropometric indices were similar between the intervention and control arms for both age groups. Prespecified exploratory analysis to investigate the effect of intervention according to sex, ethnicity, child enroled in preschool, maternal literacy, household socioeconomic status, and whether the child had been stunted in 2014 found no evidence that the effect of intervention differed in these subgroups of children (data not shown).

**Table 2a:** Health outcomes in 3‐year‐old children evaluated after 3 years of intervention, July 2016 (*n* = 1052).

	Summary statistics			
	Intervention^a^	Control^a^	Intervention effect for intervention versus control^a^			
Three‐year olds	(*N* = 538)	(*N* = 514)				
Health outcomes	% (*n*/*N*)	% (*n*/*N*)	Crude odds ratio 95% CI	*p* value	Adjusted odds ratio^b^ 95% CI	*p* value
Anaemia: Haemoglobin (Hb) < 110 g/L (primary outcome)	57.6% (310/538)	60.1% (309/514)	0.89 0.63–1.26	0.517	0.84 0.59–1.21	0.352
Moderate‐to‐severe anaemia: Hb < 100 g/L	27.1% (146/538)	31.3% (161/514)	0.80 0.52–1.21	0.283	0.70 0.47–1.04	0.081
Stunting: < − 2SD height‐for‐age *Z* score	25.2% (131/519)	22.6% (113/500)	1.16 0.72–1.88	0.547	1.13 0.69–1.84	0.634
Underweight: < − 2SD weight‐for‐age *Z* score	15.0% (78/519)	13.4% (67/500)	1.14 0.73–1.76	0.564	1.07 0.68–1.69	0.753
Acute malnutrition: < − 2SD weight‐for‐height *Z* score	4.2% (22/519)	4.8% (24/500)	0.87 0.46–1.64	0.666	0.83 0.44–1.59	0.580
Malaria infection:^c^ the presence of trophozoites and/or sporozoites, all *Plasmodium* species	29.0% (155/534)	26.5% (136/513)	n/a	n/a	n/a	n/a
Malaria infectiousness:^c^ the presence of gametocytes, all *Plasmodium* species	11.2% (60/534)	12.3% (63/513)	n/a	n/a	n/a	n/a

	Mean (SD)	Mean (SD)	Crude difference 95% CI	*p* value	Adjusted difference^b^ 95% CI	*p* value
Haemoglobin (g/L)	105.9 (13.8)	104.4 (14.6)	1.5 −1.0–4.0	0.243	1.9 −5.4–4.3	0.129
Serum ferritin (geometric mean, µg/L)	71.52 (2.14)	69.41 (2.25)	0.03 −0.11–0.17	0.663	0.02 −0.12–0.16	0.771
Height‐for‐age *Z* score^c^	−0.89 (1.67)	−1.02 (1.38)	n/a	n/a	n/a	n/a
Weight‐for‐age *Z* score^c^	−0.82 (1.17)	−0.92 (1.02)	n/a	n/a	n/a	n/a
Weight‐for‐height *Z* score^c^	−0.44 (0.91)	−0.48 (0.97)	n/a	n/a	n/a	n/a
BMI^d^‐for‐age *Z* score^c^	−0.35 (0.92)	−0.40 (0.98)	n/a	n/a	n/a	n/a
*Plasmodium* parasite density among infected children^c^ (geometric mean, parasites/μL)	497.70 (15.49)	992.27 (14.15)	n/a	n/a	n/a	n/a

Abbreviations: BMI, body mass index; CI, confidence interval; MNP, micronutrient powder; SMC, seasonal malaria chemoprevention.

^a^ By the time of the survey in 2016, children resident in intervention villages were eligible to have received MNP annually for 3 years as well as SMC for three malaria seasons, whereas children resident in control villages would only have received SMC for two malaria seasons through the national campaign.

^b^ Adjusted for sex, *Plasmodium* infection, language spoken in the home, maternal literacy and wealth quintile. All analyses account for clustering within villages.

^c^ Data on additional outcomes were not subject to statistical tests.

^d^ Body mass index.

**Table 2b:** Health outcomes in 5‐year‐old children evaluated after 3 years of intervention, July 2016 (*n* = 1081).

	Summary statistics			
	Intervention^a^	Control^a^	Intervention effect for intervention versus control^a^			
Five‐year olds	(*N* = 530)	(*N* = 551)				
Health outcomes	% (*n*/*N*)	% (*n*/*N*)	Crude odds ratio 95% CI	*p* value	Adjusted odds ratio^b^ 95% CI	*p* value
Anaemia: Haemoglobin (Hb) < 110 g/L (primary outcome)	51.3% (272/530)	53.0% (292/551)	0.91 0.60–1.38	0.643	0.90 0.60–1.35	0.607
Moderate‐to‐severe anaemia: Hb < 100 g/L	22.1% (117/530)	22.7% (125/551)	0.95 0.63–1.45	0.826	0.94 0.64–1.40	0.770
Stunting: < − 2SD height‐for‐age *Z* score	21.8% (115/528)	22.2% (121/546)	1.00 0.65–1.53	0.998	0.84 0.54–1.30	0.422
Underweight: < − 2SD weight‐for‐age *Z* score	21.8% (115/528)	18.5% (101/546)	1.24 0.87–1.77	0.230	1.01 0.72–1.42	0.934
Acute malnutrition: < − 2SD BMI‐for‐age *Z* score	7.0% (37/528)	7.9% (43/546)	0.88 0.55–1.40	0.591	0.84 0.51–1.37	0.478
Malaria infection:^c^ the presence of trophozoites and/or sporozoites, all *Plasmodium* species	37.4% (198/529)	38.6% (212/549)	n/a	n/a	n/a	n/a
Malaria infectiousness:^c^ the presence of gametocytes, all *Plasmodium* species	17.0% (90/529)	17.9% (98/549)	n/a	n/a	n/a	n/a

	Mean (SD)	Mean (SD)	Crude difference 95% CI	*p* value	Adjusted difference^b^ 95% CI	*p* value
Haemoglobin (g/L)	108.0 (13.1)	107.8 (12.7)	0.2 −2.3–2.7	0.884	0.2 −2.1–2.5	0.869
Serum ferritin (geometric mean, µg/L)	90.02 (2.16)	74.44 (2.03)	0.18 0.05–0.31	0.006	0.18 0.07–0.30	0.002
Height‐for‐age *Z* score^c^	−1.11 (1.43)	−0.99 (1.39)	n/a	n/a	n/a	n/a
Weight‐for‐age *Z* score^c^	−1.15 (1.10)	−1.12 (1.08)	n/a	n/a	n/a	n/a
BMI‐for‐age *Z* score^c^	−0.62 (0.91)	−0.70 (0.96)	n/a	n/a	n/a	n/a
*Plasmodium* parasite density among infected children^c^ (geometric mean, parasites/μL)	1224.15 (11.94)	982.40 (6.05)	n/a	n/a	n/a	n/a

Abbreviations: BMI, body mass index; CI, confidence interval; MNP, micronutrient powder; SMC, seasonal malaria chemoprevention.

^a^ By the time of the survey in 2016, children resident in intervention villages were eligible to have received MNP annually for 3 years as well as SMC for three malaria seasons, whereas children resident in control villages would only have received SMC for two malaria seasons through the national campaign.

^b^ Adjusted for sex, *Plasmodium* infection, language spoken in the home, maternal literacy and wealth quintile. All analyses account for clustering within villages.

^c^ Data on additional outcomes were not subject to statistical tests.

### 3.6 Effect of the Intervention on Cognitive Function After 3 Years of Intervention

There was no evidence of an intervention effect on children's performance in any of the tests of cognitive‐linguistic and literacy‐related skills evaluated after 3 years of intervention, with similar mean test scores recorded between children of similar age assessed in intervention and control villages; neither in the 3‐year nor the 5‐year‐olds assessed in 2016 (Tables 3a and 3b).

**Table 3a:** Cognitive outcomes in 3‐year‐old children evaluated after 3 years of intervention, May–June 2016 (*n* = 1081).

		Summary statistics			
		Intervention^a^	Control^a^		
Three‐year olds		(*n* = 543)	(*n* = 538)	Intervention effect^b^ intervention versus control	
Cognitive outcome measures	Skill being assessed	Mean (SD)	Mean (SD)	Crude difference bootstrap 95% CI	Adjusted difference^d^ bootstrap 95% CI
Visual search (number correct, max 33)	Sustained attention, executive function	22.5 (9.5)	21.9 (8.6)	0.78 −1.14–2.70	0.22 −1.72–2.14
Visual search (number of errors)	As above	3.5 (4.5)	3.8 (4.8)	−0.35 −1.00–0.34	−0.20 −0.87–0.45
Expressive vocabulary (number of words—total across two categories)	Verbal fluency, executive functioning (indicator of early literacy skills)	7.3 (3.1)	6.7 (2.9)	0.60 −0.03–1.21	0.51 −0.09–1.16
Rapid automised naming (RAN) time (15 objects) (seconds; total across two trials)	Composite indicator of early literacy skills—memory, executive function and verbal fluency	118.2 (58.2)	118.4 (58.8)	−0.93 −13.0–9.9	0.31 −11.91–12.07

Abbreviations: CI, confidence interval; MNP, micronutrient powder; SMC, seasonal malaria chemoprevention.

^a^ By the time of the survey in 2016, children resident in intervention villages were eligible to have received MNP annually for 3 years as well as SMC for three malaria seasons, whereas children resident in control villages would only have received seasonal malaria chemoprevention for two malaria seasons through the national campaign.

^b^ Data were analysed using linear mixed models with a random effect of the village to account for clustering within the community. Since the outcome data was not normally distributed, the bootstrap method was used (2000 replications) and bias corrected, bootstrap 95% confidence intervals are reported.

^c^ Digit span, mixed instructions and head, shoulders, knees and toes tasks were not administered to the three‐year‐old children.

^d^ Fully adjusted analyses control for sex, language spoken in the home, enrolment in preschool, maternal literacy and wealth quintile.

**Table 3b:** Cognitive outcomes in 5‐year‐old children evaluated after 3 years of intervention, May–June 2016 (*n* = 1115).

		Summary statistics			
		Intervention^a^	Control^a^		
Five‐year olds		(*n* = 551)	(*n* = 564)	Intervention effect^b^ intervention versus control	
Cognitive outcome measures^a^	Skill being assessed	Mean (SD)	Mean (SD)	Crude difference bootstrap 95% CI	Adjusted difference^c^ bootstrap 95% CI
Visual search (number correct, max 33)	Sustained attention, executive function	26.9 (7.1)	26.7 (7.1)	0.27−1.16–1.78	0.07−1.26–1.45
Visual search (number of errors)	As above	2.5 (5.2)	2.4 (4.6)	0.07−0.56–0.66	−0.06−0.69–0.61
Mixed instructions (number correct, max 6)	Executive function, behavioural inhibition	5.6 (1.0)	5.5 (1.1)	0.05−0.10–0.19	0.05−0.10–0.20
Heads, shoulders, knees and toes (total score)	Executive function, behavioural inhibition	26.4 (12.2)	27.7 (12.0)	−1.34−3.09–0.37	−1.26−3.08–0.67
Digit span	Sustained attention, short‐term memory	2.7 (0.76)	2.8 (0.83)	−0.09−0.22–0.04	−0.07−0.22–0.06
Expressive vocabulary (number of words—total across two categories)	Verbal fluency, executive function (indicator of early literacy skills)	8.9 (3.2)	8.8 (3.4)	−0.01−0.76–0.64	0.07−0.65–0.70
Rapid automised naming (RAN) time (24 objects) (seconds; total across two trials)	Composite indicator of early literacy skills—memory, executive function and verbal fluency	136.5 (61.3)	137.3 (56.8)	−0.61−11.53–10.49	−2.32−13.47–9.36

Abbreviations: CI, confidence interval; MNP, micronutrient powder; SMC, seasonal malaria chemoprevention.

^a^ By time of survey in 2016, children resident in intervention villages were eligible to have received MNP annually for 3 years as well as SMC for three malaria seasons, whereas children resident in control villages would only have received seasonal malaria chemoprevention for two malaria seasons through the national campaign.

^b^ Data were analysed using linear mixed models with a random effect of the village to account for clustering within the community. Since the outcome data was not normally distributed, the bootstrap method was used (2000 replications) and bias corrected, bootstrap 95% confidence intervals are reported.

^c^ Fully adjusted analyses control for sex, language spoken in the home, enrolment in preschool, maternal literacy and wealth quintile.

### 3.7 Implementation Costs of MNP Intervention

The MNP intervention (four monthly rounds of distribution) was estimated to cost 7 USD (4226 Franc CFA) per child supplemented per annum, with the costs of training, transport and personnel being the most costly items of expenditure. Sensitivity analysis explored the potential effect of variation in the price of MNP and cost of transportation; a variation in MNP price of ± 25% would result in the total cost of MNP intervention increasing or decreasing by ± 7.1%, whilst a change in transport costs of ± 12% would result in the total cost of MNP intervention changing by ± 0.09%. Since there were no significant differences in health outcomes between the two randomised arms, a cost‐effectiveness analysis was not conducted.

## 4 Discussion

This trial of MNP combined with malaria chemoprevention in children aged 3–59 months in the Sikasso region, in Southern Mali, did not reduce anaemia among 3‐ and 5‐year‐old children assessed after 1 and 3 years of intervention. Nor did the intervention have an effect on stunting or cognitive function. The prevalence of anaemia remained high, still exceeding 50% in both age groups after 3 consecutive years of intervention, with no substantive difference between the intervention and control arms. In 2014 (before SMC was scaled up across both arms), a marked difference in malaria parasitaemia was observed between children who received both SMC and MNP versus children who received neither, showing that SMC was effective at reducing the risk of *Plasmodium* infection; however, this did not translate into an impact on anaemia. After 2 further years of both interventions, the prevalence of anaemia also remained unchanged. Although the prevalence of stunting did drop substantially between 2014 and 2016, the decrease was similar for both intervention and control villages and thus unlikely to be a result of the intervention. Indeed, these trends are consistent with demographic health surveys in the Sikasso region over the same period, which also recorded a drop in stunting in children aged 6–59 months from 41% to 32% between 2012 and 2018 (INSTAT 2014, 2018).

To our knowledge, the present study is the only trial to date to evaluate the added impact of MNP in children receiving malaria chemoprevention on nutrition, health and child development outcomes. It is also unique as it evaluated the short (1 year) and longer‐term (3 years) impact of MNP, focusing first on the combined impact of MNP and SMC (2014 survey) and then the impact of MNP only (2016 survey), in two preschool age groups (3‐ and 5‐year olds), in an under‐researched part of the world with one of the highest burdens of anaemia and malaria. The 2016 survey was also able to separate the impact of MNP from the combined effect of SMC and MNP evaluated in 2014.

The null result from both surveys (2014 and 2016) was unexpected. Previous malaria and iron supplementation trials amongst school children in the same region had been highly effective, particularly the malaria intervention, where a single annual round of chemoprevention reduced the risk of anaemia by more than 40% and significantly improved children's cognitive function (Hall et al. 2002; Clarke et al. 2017). Thus, combining MNP with two rounds of SMC, a highly effective malaria control intervention, which alone has been shown to reduce the risk of anaemia in under‐fives by 44% (WHO 2012), would be expected to improve health outcomes in this younger age group too. Furthermore, the high prevalence of anaemia across both age groups in 2014 and 2016, exceeding the prevalence of *Plasmodium* infection, would suggest iron deficiency is a major contributory cause of anaemia in this region, and thus that daily MNP with iron should have some potential to reduce the risk of anaemia, but this was not observed.

Faced with a null result, it is necessary to consider the limitations of the study design and whether either the implementation of the intervention could have been insufficient to achieve the desired impact or whether the trial design and evaluation methodology were deficient in recording the effect. However, we are confident that neither of these can fully explain our findings.

First, with regard to the implementation of the intervention, all indicators suggest that the integrated delivery approach combining SMC and MNP was effective and well received (see Roschnik et al. 2019 for more details). Reported MNP coverage exceeded 80%, adherence to the MNP home fortification protocol was 65%, and acceptability was high, with 98% of parents wanting to continue giving MNP to their children. However, it is still possible that actual coverage and adherence were less than reported by the parents, as found in Kenya, where adherence as assessed by self‐report or sachet counts exceeded adherence when assessed using an electronic monitoring device (Teshome et al. 2018).

Second, in terms of the evaluation, this cluster‐randomised trial had a good balance in the characteristics of the children surveyed in 2016 to exclude participation bias as an explantion for the observed result. The characteristics of children surveyed in 2014 and 2016 were similar and would not suggest any major change in the study population over time. Cross‐contamination is a potential concern as 18%–21% of caregivers in the control arm reported having “ever” added MNP to their child's food, and we discovered that another NGO had distributed MNP to children aged 6–23 months in a subset of villages, between October 2014 and October 2015. However, since this other distribution only targeted children under 2 years and occurred after the 2014 survey and more than 8 months before the 2016 survey, it is unlikely to have had much effect on the health and nutrition outcomes of 3‐year olds and certainly not of 5‐year olds. The biomedical survey in 2016 took longer than originally planned, resulting in some communities being surveyed once the malaria transmission season had already started. Since the order in which villages were surveyed was randomised, this delay should not have introduced any bias. However, the increased risk of malaria‐related anaemia in the study population and the longer time lag between the last MNP and haemoglobin measurement could both potentially reduce the measurable effect of MNP. Nonetheless, all outcome measurements were remarkably similar between the two trial arms, and it is improbable that the delay of a few weeks can fully explain this lack of a difference.

Three recent meta‐analyses have concluded that MNP reduces the risk of iron deficiency and anaemia in children (De‐Regil et al. 2017; Tam et al. 2020; Suchdev et al. 2020). However, it is important to note that not all studies recorded an effect. A few studies from low‐income settings across Africa, Asia and Latin America, including one other MNP study in Mali (Somassè et al. 2018), only recorded marginal or no effect of MNP on anaemia, suggesting that in some contexts, MNP may not be effective (Somassè et al. 2018; Osei et al. 2015; Andrew et al. 2016; Teshome et al. 2017; Barffour et al. 2019; Ford et al. 2020). Some of these trials reported shortcomings in implementation, such as low compliance with MNP, misuse (adding to hot food affecting the MNP composition or too much food, limiting the amount consumed by the child) (Somassè et al. 2018; Osei et al. 2015; Teshome et al. 2017), or insufficient iron in the MNP (Barffour et al. 2019). Other studies identified possible biochemical or biomedical reasons for the lack of effect, including inflammation and high levels of phytates in the food vehicle reducing iron absorption (Teshome et al. 2017), cooking with soda ash, which may affect MNP bioavailability (Ford et al. 2020) or the age of children receiving MNP (over 1 year) if iron deficiency is no longer the main cause of anaemia (Osei et al. 2015; Andrew et al. 2016). We cannot exclude the possibility that we too may have missed observing intervention effects in very young children since health outcomes were only measured in children aged 3 and 5 years; it is also possible that similar biological‐related explanations may have prevented children from fully benefitting from the MNP in Mali. Consideration should thus be given to the main food vehicle (“la bouillie”), which was selected and promoted as an MNP vehicle to improve adherence since it is routinely given to children in an individual cup every morning, and which 96% of parents reported they added the MNP to (Roschnik et al. 2019). This “bouillie” is a cereal‐based meal (maize, sorghum, pearl millet) with potentially high levels of phytate, which could prevent MNP absorption (Koreissi‐Dembélé et al. 2013). Furthermore, if chemical agents such as soda ash (sodium carbonate) had been added to the bouillie to reduce cooking time, save fuel or improve taste, as reported in Uganda (Ford et al. 2020), these too could reduce the bioavailability of the iron element of the MNP. Neither of these aspects was measured during our study, and thus, we cannot exclude these as potential reasons for the lack of impact.

A second consideration is whether high levels of infection and inflammation, a potential contributory cause of anaemia, might prevent children from benefitting fully from the MNP (Mwangi et al. 2021; Muriuki et al. 2021). Sikasso region has one of the highest levels of malaria globally, with over 47% of children under five infected when the study started (INSTAT 2014). Other parasitic infections such as intestinal worms (especially hookworm) may also cause anaemia, although decades of routine deworming have reduced the prevalence of schistosomiasis and intestinal helminths to less than 2% (DNS 2019). Interventions (like SMC) that halve the risk of malaria are alone estimated to reduce the prevalence of iron deficiency (and thus anaemia) by 49% (Muriuki et al. 2021). Though SMC was given to children in this trial before MNP to clear infection with malaria parasites, 29% of 3‐year olds and 37% of 5‐year olds were still infected with *Plasmodium* parasites at endline, potentially continuing to cause anaemia. Furthermore, in 2014, when SMC reduced *Plasmodium* infection from 55% to 32% in 5‐year olds (Figure 3) little effect was observed on anaemia (54% vs. 52%), indicating that other causes of anaemia should be considered. Iron absorption may be reduced through the hepcidin hormone to prevent the harmful effects of iron on infection (Prentice 2017). There is a growing body of evidence that environmental enteric dysfunction (EED)—chronic intestinal inflammation and altered permeability—may also be a major cause of malnutrition by limiting the absorption of nutrients in the gut. EED is thought to be caused by continuous faecal–oral exposure to enteric pathogens, and, like diarrhoea, is associated with poor sanitation and hygiene in low‐income countries (Korpe and Petri 2012; Mondal et al. 2019). In Sikasso, the region where this trial was conducted, 3 in 10 households do not have access to clean drinking water, and fewer than 1 in 10 households have basic hand washing facilities (Benedict et al. 2019). Although there have been no studies of EED in southern Mali, the proportion of children under 5 years who had a diarrhoea episode in the previous 2 weeks was 9% in 2013 and 10% in 2018 (INSTAT 2014, 2018).

In conclusion, while there were some research and implementation‐related factors that may have partially contributed to the null findings in this trial, these are not sufficient to explain a complete lack of impact after 3 consecutive years of implementation in a population of children with over 60% anaemia. The implementation data available indicate that the delivery approach was broadly effective, and we contend that the biological factors discussed above are the most likely reasons for the null findings. These null findings have some important implications for global policy and research, as well as guidance on MNP and micronutrient supplementation, particularly in light of other recent evidence of the limited effect of MNP in certain contexts. First, these findings emphasise the need for more research on the root causes of anaemia as well as micronutrient effectiveness trials across a wider range of contexts, particularly settings with a high burden of infectious disease. Second, they underscore the importance of revisiting global guidance on MNP, acknowledging that MNP is not effective everywhere and that formative research to optimise the intervention for local contexts may be needed before scale‐up. We further recommend that all formative research should routinely include biological studies to assess MNP absorption and bioavailability, taking account of local diets and disease risks. Third, we recommend that future MNP interventions in low‐income settings should always include efforts to address underlying infections, such as EED, diarrhoea and malaria, alongside (or before) micronutrient supplementation, to yield the full potential of nutritional interventions to address the persistent high levels of anaemia.

## Author Contributions

S.E.C., M.S. and N.R. formulated the research questions and designed the trial. N.H.D., Y.D. and N.R. developed the interventions and led the implementation of the interventions in study villages. All authors participated in the development and piloting of assessment tools and the conducting of one or more of the final evaluation surveys. M.S. and R.S. conducted biomedical surveys and laboratory analyses. Y.G. and L.P. designed and interpreted the analyses of cognitive and child development tests. J.T. and H.T. designed, implemented and analysed the costing data. Statistical analyses were conducted by R.J., with additional advice from M.B. and H.V. on assessment methodology and interpretation of results. Support in data management, data analysis and preparation of the final manuscript was provided by S.L., K.S. and H.M. All authors read and approved the final manuscript.

## Ethics Statement

This study was conducted according to the guidelines laid down in the Declaration of Helsinki and all procedures involving research study participants were approved by the Comite d'Ethique de l'INRSP, Ministry of Health, Mali and LSHTM ethics committee, UK. Written informed consent was obtained from all subjects/patients. In July 2013, community meetings were held with parents and local community representatives to explain the purpose of the study and procedures to be followed (including randomisation of communities), after which communities were offered the choice to participate in the trial. Community meetings were repeated in May 2014 and 2016 to obtain written informed consent from the parents of each child selected to participate in the surveys. Signed consent before the interview was also obtained from all individuals interviewed during the formative research and qualitative evaluation. To safeguard child rights, all project staff and survey team members were oriented on and signed the Save the Children's child safety policy.

## Conflicts of Interest

The authors declare no conflicts of interest.

## Supporting information

Mali SIEF MNP outcomes paper Supporting material 10 3 25 clean (1).

## Data Availability

The data that support the findings of this study are openly available in the World Bank databank at https://databank.worldbank.org.
