# Supplementary material for: Micronutrient Powders Combined With Malaria Chemoprevention to Improve Anaemia and Cognitive Function in Early Childhood in Mali: A Cluster‐Randomised Trial
Source: Matern Child Nutr. 2025 May 20;21(4):e70033. doi: 10.1111/mcn.70033 (PMC12454176; doi:10.1111/mcn.70033)
Supplement: Supplementary file 1 — Mali SIEF MNP outcomes paper Supporting material 10 3 25 clean (1). [file MCN-21-e70033-s001.docx]

Supplementary Table S1. **Description of cognitive tests which were used in each age group**

| **Name of test** | **Cognitive skills assessed** | **3-year olds** | **5-year**  **olds** | **References** |
| --- | --- | --- | --- | --- |
| Visual search | Sustained attention,  Executive function | ✓ | ✓ | Adapted from: Gooch D, Thompson P, Nash HM, Snowling MJ & Hulme C. The development of executive function and language skills in the early school years. *Journal of Child Psychology and Psychiatry*, 2016, 57(2), 180-187. |
| Mixed instructions | Executive function  (specifically behavioural inhibition) | ✓ | ✓ | Adapted from: Diamond A & Taylor C. Development of an aspect of executive control: development of the abilities to remember what I said and to “Do as I say, not as I do”. *Dev. Psychobiol*. 1996, 29, 315–334.  doi: 10.1002/(SICI)1098-2302(199605)29:4<315::AID-DEV2<3.0.CO;2-T |
| Head, Shoulders, Knees and Toes | Executive function;  Behavioural inhibition | n/a | ✓ | Adapted from: Ponitz CE, McClelland MM, Jewkes AM, Connor CM, Farris CL & Morrison FJ. Touch your toes! Developing a direct measure of behavioral regulation in early childhood. *Early Childhood Research Quarterly*, 2008, 23(2), 141-158. <http://doi.org/10.1016/j.ecresq.2007.01.004> |
| Forward digit span | Sustained attention,  Verbal short-term memory | n/a | ✓ | Adapted from IDELA; Pisani L, Borisova I & Dowd A. Developing and validating the International Development and Early Learning Assessment (IDELA). *International Journal of Educational Research,* 2018, 91*,* 1-15. <https://doi.org/10.1016/j.ijer.2018.06.007> |
| Expressive vocabulary | Verbal fluency,  Executive functioning (indicator of early literacy skills) | ✓ | ✓ | Adapted from IDELA; Pisani L, Borisova I & Dowd A. Developing and validating the International Development and Early Learning Assessment (IDELA). *International Journal of Educational Research,* 2018, 91*,* 1-15. <https://doi.org/10.1016/j.ijer.2018.06.007> |
| Rapid automised naming [RAN] | Composite indicator of early literacy skills:  memory, executive function, verbal fluency | ✓  15 objects | ✓  24 objects | Adapted from: Caravolas M, Lervåg A, Mousikou P, Efrim C, Litavský M, Onochie-Quintanilla E, Salas N, Schöffelová M, Defior S, Mikulajova M, Málková G & Hulme C. Common patterns of prediction of literacy development in different alphabetic orthographies. *Psychological science*, 2012, *23*(6), 678-686. |

Table S2. **Comparison of characteristics of children re-surveyed and those no longer resident in the study villages in 2016**^†^

Of the 1,577 children aged 3 years at the time of the surveys in 2014(group 2), a total of 1,437 (91%) were successfully contacted and re-surveyed in 2016; now aged 5 years old. Data on household characteristics were collected through a questionnaire interview with the child’s primary caregiver in May 2014, with data from parental interviews available for 1,221 (77.4%) of the 3-year old children examined in the biomedical and/or cognitive surveys in June/July 2014.

| **Characteristics of children** | **Re-surveyed**  **in 2016**  **n = 1,437** | | **No longer resident in study villages**  **n = 140** | |  |
| --- | --- | --- | --- | --- | --- |
|  | % | n / N | % | n / N | *p*-value |
| - **Male child** | 52.8 | 759/1,437 | 53.6 | 75/140 | 0.835 |
| **Parent questionnaire completed in 2014** | 77.8 | 1,118/1,437 | 73.6 | 103/140 | - |
| **Main language spoken in the home**   - Bambara - Shenara - Mamara - French - Other | 37.8  47.5  10.9  1.3  2.5 | 384/1,016  483/1,016  111/1,016  13/1,016  25/1,016 | 36.6  48.4  11.8  1.1  2.2 | 34/93  45/93  11/93  1/93  2/93 | 0.994 |
| **Mother is literate** | 12.2 | 116/949 | 16.1 | 15/93 | 0.270 |
| **Father attended school** | 23.3 | 218/935 | 21.8 | 19/87 | 0.448 |
| **Income:** Subsistence agriculture | 93.9 | 1,050/1,118 | 94.1 | 95/101 | 0.952 |
| **Standard of housing :**   - Metal roof, tile or concrete - Tiles/concrete floor - Brick/concrete walls | 79.2  23.5  3.1 | 753/951  262/1,115  35/1,118 | 80.9  25.5  7.8 | 68/84  26/102  8/103 | 0.651  0.653  0.007 |
| **Solar/electric lighting** | 66.7 | 725/1,087 | 71.7 | 71/99 | 0.309 |
| **Wealth index (household assets)**   - 1^st^ quartile (most poor) - 2^nd^ quartile - 3^rd^ quartile - 4^th^ quartile (least poor) | 24.7  23.4  20.4  31.5 | 249/1,007  236/1,007  205/1,007  317/1,007 | 18.3  30.1  19.4  32.3 | 17/93  28/93  18/93  30/93 | 0.372 |
| **Child enrolled in ECD centre in 2014**^‡^ | 14.4 | 158/1,101 | 12.6 | 13/103 | 0.178 |

^†^ Data are not presented for children aged 5 years at the time of the survey in 2014, as these children were not re-surveyed in 2016. Neither are data available for the youngest cohort (aged <1 year at the start of the trial; aged 3 years in 2016) since parental interviews, biomedical and cognitive surveys were not carried out in this age group in 2014;

^‡^This cohort of children were aged 3 years in 2014, and few are enrolled in an ECD centre by this age.

Table S3. **Characteristics of foods eaten, home literacy environment and parent-child interactions reported by 3y and 5y children’s parents/caregivers during household surveys in 2016, by study arm**

|  | **Intervention** | **Control** |
| --- | --- | --- |
|  | (30 villages) | (30 villages) |
| **Interventions** | **MNP +**  **Parenting sessions** | **Parenting sessions only**^†^ |
| **Nutritional practices^2^** | % | % |
| *Proportion of caregivers reporting that in the day prior to survey:*   - Child had eaten at least 4 times [snacks and meals] | 67.4 | 66.1 |
| - Child had consumed cereals and grains | 99.7 | 99.5 |
| - Child had consumed root vegetables [cassava, potatoes, yams] | 26.2 | 23.6 |
| - Child had consumed beans and nuts | 67.0 | 61.1 |
| - Child had consumed other vegetables | 23.5 | 20.6 |
| - Child had consumed fruit | 96.2 | 96.0 |
| - Child had consumed meat, poultry or fish | 68.2 | 71.5 |
| - Child had consumed milk | 36.9 | 32.1 |
| - Child had consumed eggs | 18.6 | 18.8 |
| - Child had consumed palm oil | 12.9 | 9.9 |
| - Child had consumed other fats and oils | 93.9 | 94.7 |
| **Home literacy environment**^‡^ | Mean (SD) | Mean (SD) |
| Number of reading materials in the home | 1.95 (1.99) | 1.79 (1.94) |
| Number of toys and other play items in the home | 3.76 (1.41) | 3.69 (1.35) |
| **Adult-Child Interaction**^‡^ | % | % |
| *Proportion of caregivers reporting that in the last week a parent or other family member had:*  - Played with child | 95.2 | 95.0 |
| - Hugged or showed affection to child | 92.9 | 92.5 |
| - Taken child on visit outside the home | 77.1 | 80.2 |
| - Sung song or lullaby to the child | 75.3 | 72.7 |
| - Named objects for the child | 63.8 | 64.0 |
| - Told stories to the child | 59.3 | 55.4 |
| - Shown or taught something new to child | 53.0 | 46.7 |
| - Played counting game or taught numbers | 51.2 | 48.0 |
| - Read or looked at books with child | 36.1 | 31.1 |
| - Drawn something for /with child | 34.9 | 30.6 |
| - Taught alphabet or letters to child | 34.6 | 29.5 |

^†^Although both groups of villages organised parent information sessions which included key messages on child nutrition, the cooking demonstrations were only held in MNP intervention villages.

^‡^ To minimise statistical concerns of multiple comparisons, data were not subject to formal statistical testing; but are reported here for completeness. The marginally higher dietary diversity seen in MNP intervention villages could indicate that cooking demonstrations were a useful addition to the informational meetings (both in terms of providing practical examples based on locally available foodstuffs and reinforcing verbal messaging), and/or that there had been greater focus on child nutrition during the information sessions in these villages (frequency and intensity of communication), however it is not possible to fully separate these two effects.

Table S4. **Coverage and acceptability of the SMC and MNP interventions reported by children’s parents/caregivers during household surveys in intervention villages in 2014 and 2016.**

|  | **2014 surveys**^†^  (after 1 year of MNP distribution) | | **2016 surveys**^‡^  (after 3 years of MNP distribution) | | |
| --- | --- | --- | --- | --- | --- |
|  | **n / N** | **%** | **n / N** | **%** |  |
| **Coverage of SMC intervention** |  |  |  |  |  |
| Caregiver said their child received SMC during the most recent malaria transmission season | 317 / 354 | 89.5 | 1101 / 1137 | 96.8 |  |
| **Coverage of MNP intervention** |  |  |  |  |  |
| Caregiver said they had ever added MNP to their child’s food | 369 / 453 | 81.5 | 899 / 1148 | 78.3 |  |
| Caregiver said they added MNP to their child’s food at least 4 days per week in the last 7 days | 295 / 453 | 65.1 | 755 / 1148 | 65.8 |  |
| **Acceptability of MNP intervention** |  |  |  |  |  |
| Caregiver reported that their child liked to eat the food with the MNP | N/A | N/A | 857 / 908 | 94.4 |  |
| Caregiver reported rarely or never experiencing any difficulty in giving MNP to their child | 187 / 323 | 57.9 | 835 / 904 | 92.4 |  |
| Caregiver noticed changes in their child since giving MNP | 241 / 341 | 70.7 | 824 / 855 | 96.4 |  |
| Percentage reporting a positive change  Types of changes reported:   - Child appetite increased - Child less sick than normal - Child more active/energetic than normal | 258 / 269  202 / 269  157 / 269  82 / 269 | 96.1  75.1  58.4  30.5 | 705 / 823  537 / 823  407 / 823  327 / 823 | 85.7  62.8  49.4  39.7 |  |
| Percentage reporting a negative change  Types of changes reported:   - Child more sick than normal - Child less active/energetic than normal | 40 / 269  15 / 269  25 / 269 | 15.0  5.6  9.3 | 118 / 823  46 / 823  39 / 823 | 14.3  5.6  4.7 |  |
| Caregivers wanted to give MNP the following year | 335 / 341 | 98.2 | 871 / 890 | 97.9 |  |
|  | | | | | |

^†^ Data on intervention coverage were only available from approx 1/3 of children in the intervention villages surveyed in 2014 (data loss due to a computer virus); ^‡^Household survey data was available from all children assessed in intervention villages in 2016.

Table S5a. **Health outcomes in 3-year old children evaluated after 1 year of the MNP+SMC intervention, June-July 2014 (n=1,105)**

|  | **Summary statistics** | | **Intervention Effect for**  **Intervention vs Control**^‡^ | |
| --- | --- | --- | --- | --- |
|  | **Intervention**^†^ | **Control**^†^ | **Crude**  **Odds ratio  95% CI** | **p value** |
| **Three year olds** | **(N = 550)** | **(N = 555)** |  |  |
|  | % (n / N) | % (n / N) |  |  |
| Anaemia:  Haemoglobin [Hb] <110g/L  [primary outcome] | 61.6%  (339 / 550) | 64.0%  (355 / 555) | 0.91  0.63 to 1.31 | 0.618 |
| Moderate-to-severe anaemia:  Hb <80g/L | 2.9%  (16 / 550) | 4.0%  (22 / 555) | 0.71  0.28 to 1.79 | 0.462 |
| Stunting:  <-2SD Height-for-age Z-score | 47.8%  (263 / 550) | 44.0%  (244 / 555) | 1.20 0.83 to 1.73 | 0.325 |
| Underweight:  <-2SD Weight-for-age Z-score | 26.2%  (144/ 550) | 25.4%  (141 / 555) | 1.07  0.75 to 1.53 | 0.701 |
| Acute malnutrition:  <-2SD Weight-for-height Z-score | 4.7%  (26 / 550) | 6.3%  (35 / 555) | 0.76  0.39 to 1.48 | 0.423 |
| Malaria infection  [presence of trophozoites and/or sporozoites, all *Plasmodium* species, co-primary outcome] | 21.1%  (116 / 550) | 44.5%  (247 / 555) | 0.30  0.19 to 0.47 | <0.001 |
| Malaria infectiousness^3^  [presence of gametocytes, all *Plasmodium* species] | 7.5%  (41 / 550) | 16.8%  (93 / 555) | n/a | n/a |
|  | **Mean (SD)** | **Mean (SD)** | **Difference  95% CI** | **p value** |
| Haemoglobin [g/L] | 105.64 (11.90) | 104.40 (12.70) | 1.24  -1.12 to 3.43 | 0.320 |
| Height-for-age Z-score^§^ | -1.91 (1.82) | -1.73 (1.56) | n/a | n/a |
| Weight-for-age Z-score^§^ | -1.38 (1.26) | -1.32 (1.17) | n/a | n/a |
| Weight-for-height Z-score^§^ | -0.38 (1.12) | -0.45 (1.16) | n/a | n/a |
| BMI^¶^-for-age Z-score^§^ | -0.14 (1.17) | -0.25 (1.19) | n/a | n/a |
| *Plasmodium* parasite density  among infected children^§^  [geometric mean, parasites/μL] | 512.86  (10.28) | 639.06  (7.46) | n/a | n/a |

^†^By time of survey in 2014, children resident in intervention villages were eligible to have received MNP as well as SMC for one year, whereas children resident in control villages would only have received neither. ^‡^All analyses account for clustering within villages; Due to incomplete household survey data (due to data loss resulting from a computer virus), estimates of the intervention effect could not be adjusted for sex, *Plasmodium* infection, language spoken in the home, maternal literacy, and wealth quintile, as planned. ^§^ To minimise statistical concerns of multiple comparisons only major outcomes were subject to formal statistical testing, and p-values are not available (n/a) for all outcomes, serum ferritin was not measured in 2014; ^¶^Body mass index.

Table S5b. **Health outcomes in 5-year old children evaluated after 1 year of the MNP+SMC intervention, June-July 2014 (n=1,033)**

|  | **Summary statistics** | | **Intervention Effect for**  **Intervention vs Control**^‡^ | |
| --- | --- | --- | --- | --- |
|  | **Intervention**^†^ | **Control**^†^ | **Crude**  **Odds ratio  95% CI** | **p value** |
| **Five year olds** | **(N = 522)** | **(N = 511)** |  |  |
|  | % (n / N) | % (n / N) |  |  |
| Anaemia:  Haemoglobin [Hb] <110g/L  [primary outcome] | 53.8%  (281 / 522) | 51.9%  (265 / 511) | 1.10  0.78 to 1.55 | 0.582 |
| Moderate-to-severe anaemia:  Hb <80g/L | 2.7%  (14 / 522) | 1.8%  (9 / 511) | 1.54  0.66 to 3.58 | 0.319 |
| Stunting:  <-2SD Height-for-age Z-score | 35.4%  (185 / 522) | 32.3%  (165 / 511) | 1.15 0.77 to 1.71 | 0.493 |
| Underweight:  <-2SD Weight-for-age Z-score | 26.6%  (139/ 522) | 25.0%  (128 / 511) | 1.11  0.76 to 1.61 | 0.598 |
| Acute malnutrition:  <-2SD BMI-for-age Z-score | 5.2%  (27 / 522) | 6.1%  (31 / 511) | 0.80  0.33 to 1.92 | 0.614 |
| Malaria infection  [presence of trophozoites and/or sporozoites, all *Plasmodium* species, co-primary outcome] | 32.4%  (169 / 521) | 55.0%  (281 / 511) | 0.35  0.22 to 0.57 | <0.001 |
| Malaria infectiousness^§^  [presence of gametocytes, all *Plasmodium* species] | 11.7%  (61 / 521) | 17.6%  (90 / 511) | n/a | n/a |
|  | **Mean (SD)** | **Mean (SD)** | **Difference  95% CI** | **p value** |
| Haemoglobin [g/L] | 107.08 (12.67) | 108.35 (11.59) | -1.27  -3.57 to 0.67 | 0.181 |
| Height-for-age Z-score^§^ | -1.58 (1.54) | -1.52 (1.23) | n/a | n/a |
| Weight-for-age Z-score^§^ | -1.34 (1.16) | -1.31 (1.00) | n/a | n/a |
| BMI^¶^-for-age Z-score^§^ | -0.45 (1.15) | -0.49 (0.98) | n/a | n/a |
| *Plasmodium* parasite density  among infected children^§^  [geometric mean, parasites/μL] | 727.78  (11.94) | 523.22 (6.05) | n/a | n/a |

^†^By time of survey in 2014, children resident in intervention villages were eligible to have received MNP as well as SMC for one year, whereas children resident in control villages would only have received neither. ^‡^All analyses account for clustering within villages; Due to incomplete household survey data (due to data loss resulting from a computer virus), estimates of the intervention effect could not be adjusted for sex, *Plasmodium* infection, language spoken in the home, maternal literacy, and wealth quintile, as planned. ^§^To minimise statistical concerns of multiple comparisons only major outcomes were subject to formal statistical testing, and p-values are not available (n/a) for all outcomes, serum ferritin was not measured in 2014; ^¶^Body mass index.
